# Phorbol 12-Myristate 13-Acetate Induced Toxicity Study and the Role of Tangeretin in Abrogating HIF-1α-NF-κB Crosstalk In Vitro and In Vivo

**DOI:** 10.3390/ijms21239261

**Published:** 2020-12-04

**Authors:** Sukkum Ngullie Chang, Debasish Kumar Dey, Seong Taek Oh, Won Ho Kong, Kiu Hyung Cho, Ebtesam M. Al-Olayan, Buyng Su Hwang, Sun Chul Kang, Jae Gyu Park

**Affiliations:** 1 Department of Biotechnology, Daegu University, Gyeongsan 38453, Korea; sukkumchang@gmail.com (S.N.C.); deepdey1993@daegu.ac.kr (D.K.D.); 2 Advanced Bio Convergence Center, Pohang Technopark Foundation, Pohang 37668, Gyeongbuk, Korea; isotjdxorl@nate.com (S.T.O.); whkong@ptp.or.kr (W.H.K.); 3 Okinawa Research Center Co. Ltd., 13-33, Suzaki, Uruma-si, Okinawa Ken 904-2234, Japan; 4 Research Group, Gyeongbuk Institute for Bio Industry (GIB), Andong 36728, Korea; khcho68@gmail.com; 5 Department of Zoology, Faculty of Science, King Saud University, Riyadh 11451, Saudi Arabia; 0111718192@yahoo.com; 6 Nakdonggang National Institute of Biological Resources, Sangju 37242, Korea; hwang1531@nnibr.re.kr

**Keywords:** phorbol 12-myristate 13-acetate (PMA), *allium cepa* test, zebrafish embryotoxicity test, inflammation, tangeretin (TAN), nuclear factor kappa-light-chain-enhancer of activated b cells (NF-κb), hypoxia-inducible factor 1-alpha (HIF-1α)

## Abstract

Phorbol 12-myristate 13-acetate (PMA) is a potent tumor promoter and highly inflammatory in nature. Here, we investigated the toxic effects of PMA on different model system. PMA (10 μg) caused chromosomal aberrations on the *Allium cepa* root tip and induced mitotic dysfunction. Similarly, PMA caused embryonic and larval deformities and a plummeted survivability rate on zebrafish embryo in a dose-dependent manner. Persistently, PMA treatment on immortalized human keratinocyte human keratinocyte (HaCaT) cells caused massive inflammatory rush at 4 h and a drop in cell survivability at 24 h. Concomitantly, we replicated a cutaneous inflammation similar to human psoriasis induced by PMA. Herein, we used tangeretin (TAN), as an antagonist to counteract the inflammatory response. Results from an in vivo experiment indicated that TAN (10 and 30 mg/kg) significantly inhibited PMA stimulated epidermal hyperplasia and intra-epidermal neutrophilic abscesses. In addition, its treatment effectively neutralized PMA induced elevated reactive oxygen species (ROS) generation on in vitro and in vivo systems, promoting antioxidant response. The association of hypoxia-inducible factor 1-alpha (HIF-1α)-nuclear factor kappa-light-chain-enhancer of activated b cells (NF-κB) crosstalk triggered by PMA enhanced PKCα-ERK1/2-NF-κB pathway; its activation was also significantly counteracted after TAN treatment. Conclusively, we demonstrated TAN inhibited the nuclear translocation of HIF-1α and NF-κB p65. Collectively, TAN treatment ameliorated PMA incited malignant inflammatory response by remodeling the cutaneous microenvironment.

## 1. Introduction

Tumor promoters and inflammation inducers such as phorbol esters are naturally occurring compounds referred to as tigilane diterpene [1], extracted from numerous plants such as *Jathropa curcas, Excoecaria agallocha, Homalanthus nutans, Sapium indicum, S. japonicum* [2]. The structure of phorbol ester is based upon the tetracycline diterpene carbon skeleton, also known as tigliane. Among the many different phorbol esters, phorbol 12-myristate 13-acetate (PMA) isolated from the unripe fruit of *Sapium indicum* is a highly pro-inflammatory agent and tumor promoter [3]. Numerous studies have shown that PMA can stimulate the activity of protein kinase C (PKC), involved in a molecular signaling pathway for the development of most cells and tissues with a variety of biological effects. PMA acts by releasing proteases, cytokines, and NADPH oxidases which, in turn, contribute to tissue damage or activates the production of histamines, which promote vascular remodeling, redness, heat, and tumor promotion, as observed from the topical application on the skins in murine models. Pro-inflammatory cytokines are the important radical mediators of inflammatory diseases [4], and are involved in multitudinous diseases [5]. Nuclear factor-kappa B (NF-κB), a major transcription factor involved in the activation of pro-inflammatory response, it is a crucial pathway that elevates during inflammation. NF-κB has been implicated as a major contributor to numerous human diseases [6]. Here, we focused on the translocation of NF-κB subunits (p50/p65) from the cytoplasm into the nucleus that occurs during an inflammatory response and its interaction with another transcription factor hypoxia-inducible factor 1α (HIF-1α), which is associated with numerous pathological conditions in inflammatory diseases [7]. Several studies showed that hypoxia is also involved in the activation of NF-κB pathway. Hypoxia has also been implicated as an activator of both the HIF-1α and NF-κB through the TAK-1 mediated pathway. Studies on pro-inflammatory cytokines, such as IL-1β and TNF-α, have shown that they can stabilize, activate, and recruit HIF-1α on the site of inflammation [8]. The crosstalk between HIF-1α-NF-κB is mediated by an extracellular stimuli-responsive kinase (ERK1/2), which is involved in the phosphorylation of p65 ser^276^, and also through the upregulation of a signaling cascade, phosphorylating the IκBα, leading to its degradation and the consequent p50/p65 nuclear localization involved in the transcription of pro-inflammatory cytokines and other transcriptional targets.

In this study, we assessed the toxicity of PMA on different model systems such *Allium cepa*. Zebrafish embryo, human keratinocyte (HaCaT) cells, and also studied a BALB/c mice model. Next, we used tangeretin (4′,5,6,7,8-pentamethoxy flavone) as an inflammatory antagonist in the present study. Numerous studies have shown the anticancer and anti-inflammatory potential of tangeretin alone, as well as in combination with the standard anti-cancer drugs [9,10]. We induced an inflammatory response using PMA as an inflammation inducer similar to the symptoms of human psoriasis on BALB/c mice. Hereon, our study was majorly focused on the anti-inflammatory role of tangeretin on an in vitro and in vivo system.

## 2. Results and Discussion

### 2.1. Effect of PMA on Allium cepa Root Tip

**Figure 1:**
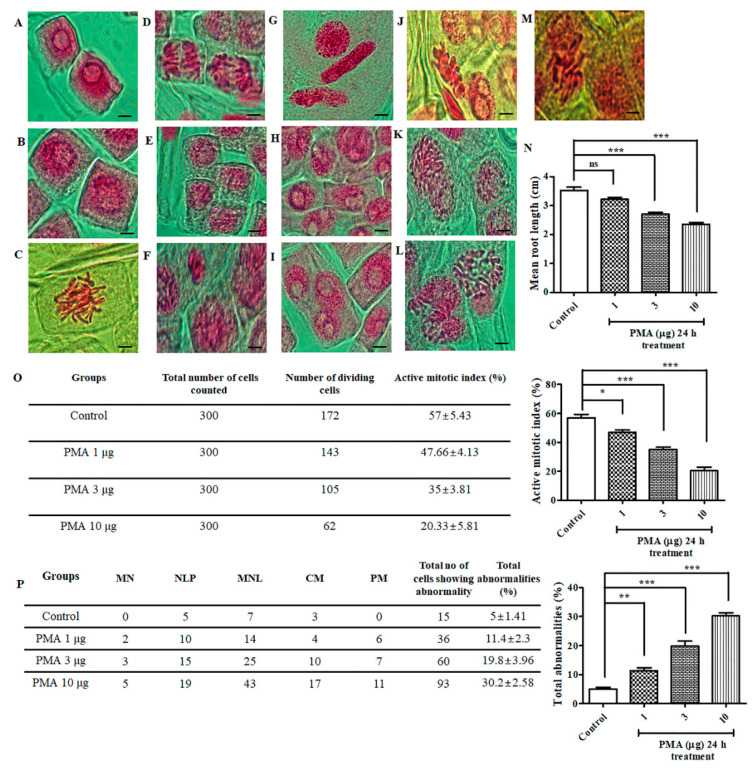
Chromosomal aberrations induced by phorbol 12-myristate 13-acetate (PMA) in the root tip meristem of *Allium cepa.* Acetocarmine stain was used for staining the root tips to observe the different stages of mitosis. (**A**–**E**) Normal stages of mitosis in onion root tip: (**A**) Interphase, (**B**) Prophase, (**C**) Metaphase, (**D**) Anaphase, (**E**) Telophase. Different aberrations were observed after PMA (0.1 μg, 1 μg, 10 μg) treatment for 24 h. (**F**) Micronucleus, (**G**) Loculated nucleus, (**H**) Nuclear lesion, (**I**) Multi-nucleated cell lesion, (**J**) C-metaphase, (**K**) Polyploidy and chromosomal bridge, (**L**) Laggard metaphase and anaphase, (**M**) Pulverized metaphase, (**N**) Mean root length of *A. cepa*, (**O**) Percentage of mitotic index, (**P**) Chromosomal aberrations and percentage of total abnormalities. Scale bar = 10 μm. The data are represented as the means ± S.D. of three independent experiments * *p* < 0.05, ** *p <* 0.01, *** *p* < 0.001 and ns (non-significant). Statistical significance analysis was carried out through a one-way analysis of variance (ANOVA) prism.

Images of different chromosomal aberrations are depicted in Figure 1F–M, in comparison to the normal mitotic stages (Figure 1A–E). For evaluating the genotoxicity of PMA on *Allium cepa* meristematic cells, the roots were exposed to varying concentration of PMA (1, 3, 10 μg). There was a significant reduction in the root length observed on the 3 μg and 10 μg doses (Figure 1N). We calculated the mitotic index (*p* < 0.0001) and observed a remarkable inhibition in different concentration of PMA exposure groups as follows: 1 μg, 3 μg and 10 μg (47.66%, 35%, 20.33%) in comparison to the control (57%) (Figure 1O). Subsequently, total abnormalities (*p* < 0.0001) were spiked with increase in PMA dose, as observed (Figure 1P). The occurrence of abnormality percentage for the respective groups were control (5%), PMA 1 μg, 3 μg, and 10 μg (11.4%, 19.8%, and 30.2% respectively). Collectively, through histological analysis, we observed that PMA exerted chromosomal abnormalities and mitotic dysfunction in *Allium cepa* model.

### 2.2. Effect of PMA on Zebrafish Embryonic Malformation

**Figure 2:**
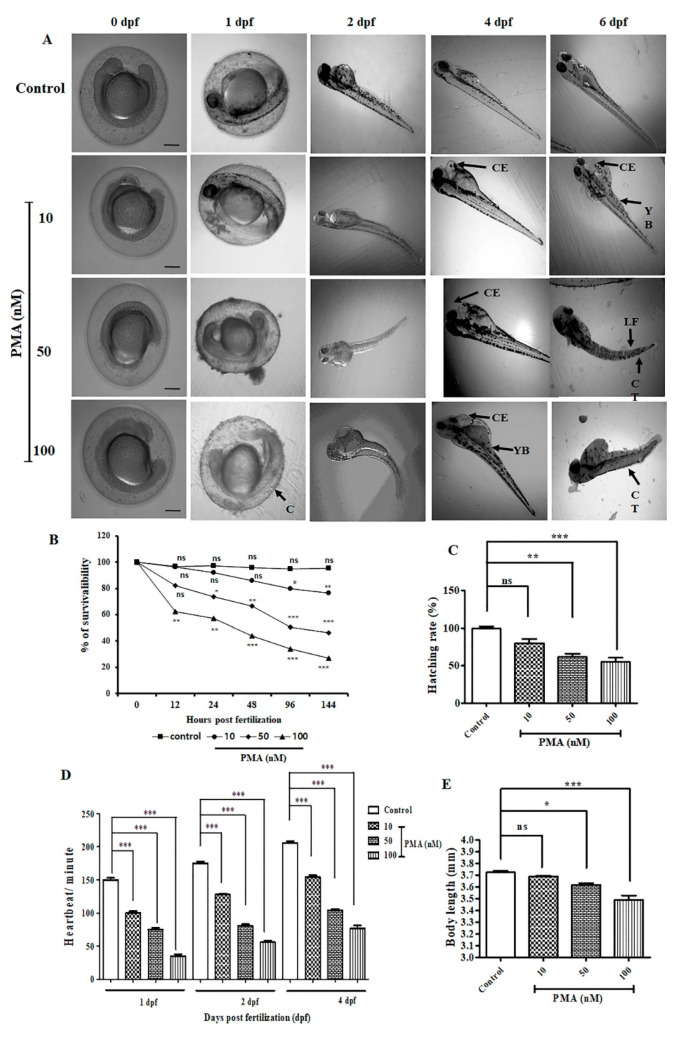
PMA exerted various deformities in zebrafish embryo and larvae (*n* = 10/group). (**A**) Morphological images of the development of zebrafish embryo to larval stage. Images were captured from 0.5–6 dpf. Common observed deformities were C (coagulation), CE (cardiac edema), CT (curved tail), LF (lack of tail fins), YB (development of yolk bags. Scale bar = 200 μM at 4× magnification. (**B**) Percentage of survivability (**C**) Percentage of hatching rate (**D**) Determination of heartbeat per minute of on 1 dpf, 2 dpf, and 4 dpf. (**E**) The data are represented as the means ± S.D. of three independent experiments * *p* < 0.05, ** *p* < 0.01, *** *p* < 0.001 and ns (non-significant). Statistical significance analysis was carried out through a one-way analysis of variance (ANOVA) prism.

To evaluate the toxic effects of PMA on zebrafish embryo, we treated different concentration of PMA (10 nM, 50 nM, and 100 nM) and assessed the morphological changes on 0.5, 1, 2, 4, and 6-days post fertilization (dpf). Common observed embryonic deformities on 1 dpf after treatment with PMA were coagulation resulting in dead embryos. Other deformities observed on 2 dpf, 4 dpf, and 6 dpf were cardiac edema, development of yolk bags, lack of tail fins and curved tail (Figure 2A), as an indication of the induction of toxic effects. The hatching rate of embryos were significantly reduced on 50 nM and 100 nM PMA treatment groups in comparison with no treatment group (Figure 2C). Survivability rate plummeted drastically in the embryos upon prolonged PMA exposure and higher PMA dose (50−100 nM) (Figure 2B). Heartbeat calculated on 1 dpf, 2 dpf, and 4 dpf revealed a dose-dependent and time dependent decrease in the number of heart beats per minute and revealed the toxic effects of PMA treatment during the development of zebrafish embryo to larval stage (Figure 2D). Furthermore, the body length (Figure 2E) was also reduced significantly in 100 nM PMA treated larvae indicating adverse larval malformation.

### 2.3. Effect of PMA on Immortalized Human Keratinocyte HaCaT Cells

**Figure 3:**
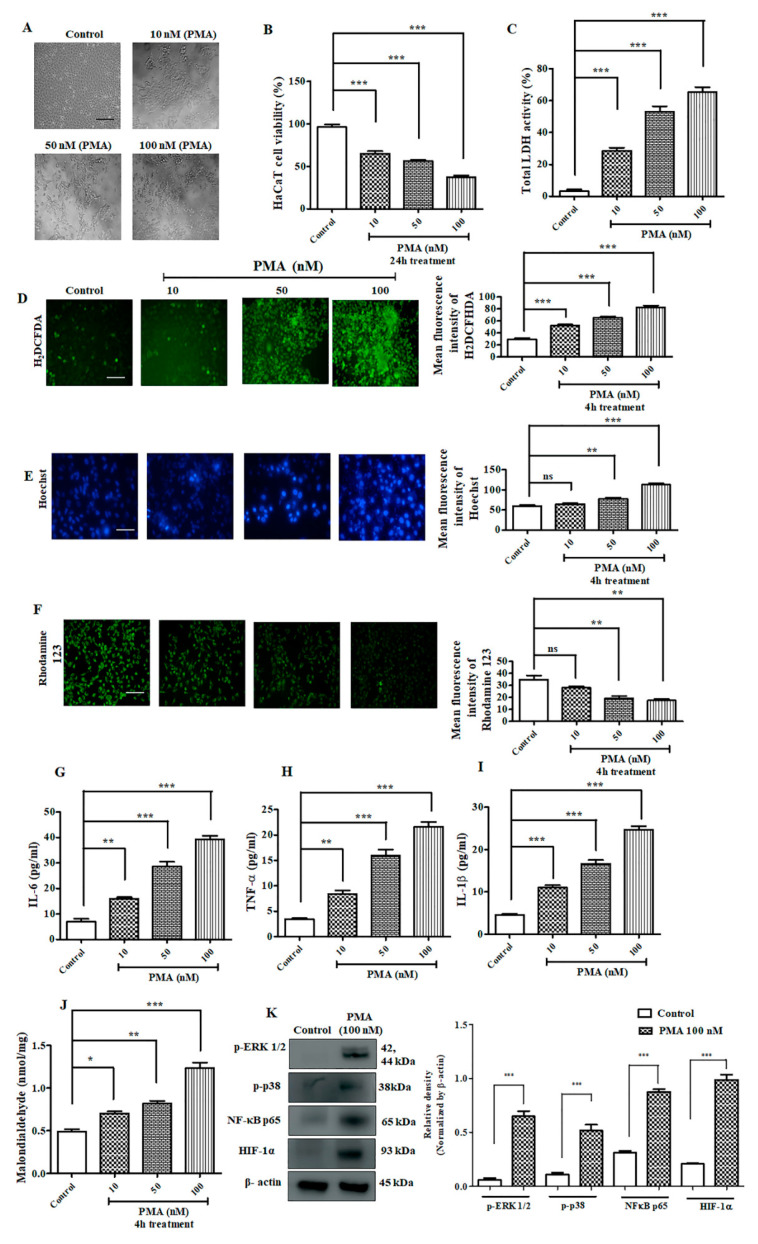
Effect of PMA on immortalized human keratinocyte (HACaT) cells. (**A**) Morphological image of HaCaT cells captured after 24 h of treatment with different concentration of PMA at different concentration (10 nM, 50 nM, 100 nM). Scale bar (100 μM), (**B**) HaCaT cell viability after 24 h PMA treatment, (**C**) LDH assay on HaCaT cells after 24 h PMA treatment, (**D**) H_2_ DCFDA staining on HaCaT cells after 4 h PMA treatment (**E**) Hoechst staining on HaCaT cells after 4 h PMA treatment, (**F**) rhodamine 123 staining on HaCaT cells after 4 h PMA treatment, (100 μM), (**G**) IL-6 estimation on HaCaT cells, (**H**) TNF-α estimation, (**I**) IL-1β estimation, (**J**) Malondialdehyde assay, (**K**) Western blotting analysis. Densitometry analysis of these respective proteins were normalized by β-actin and evaluated through Image J software. The data are represented as mean ± S.D. of three independent experiments * *p* < 0.05, ** *p* < 0.01, *** *p* < 0.001 and ns (non-significant). Control vs. PMA. Statistical significance analysis was carried out through a one-way analysis of variance (ANOVA) prism.

HaCaT cells were treated with different concentration of PMA. The morphological images of HaCaT cells (Figure 3A) captured after 24 h of treatment revealed distorted structure, DNA fragmentation, and apoptotic nuclei. The percentages of cell viability assay after PMA treatment (10 nM, 50 nM, and 100 nM) were 66.60%, 57.05%, and 36.68%, respectively (Figure 3B). The production of LDH (lactate dehydrogenase) was significantly upregulated dose-dependently (Figure 3C). On the basis of MTT and LDH results, we further carried out the remainder of experiment on HaCaT cells with PMA incubation at 4 h [11]. Next, we performed H_2_DCFDA (2′,7′-dichlorodihydrofluorescein diacetate) staining, which revealed increased intracellular reactive oxygen species (ROS) generation dose-dependently after PMA treatment (Figure 3D). In addition, Hoechst 33342 staining exhibited morphological alterations in the nucleus. Precisely, higher cell shrinkage, chromatin condensation, and nuclear fragmentation was observed upon PMA treatment in a dose-dependent manner (Figure 3E). Similarly, rhodamine 123 staining also revealed the loss of mitochondrial membrane potential with increasing dose of PMA treatment on HaCaT cells (Figure 3F). The levels of pro-inflammatory cytokines were drastically elevated dose-dependently (Figure 3G–I). We also performed MDA assay to measure the level of lipid peroxidation. As observed in Figure 3J, MDA concentration was elevated in a dose-dependent manner as shown, 0.703 nmol/mg, 0.82 nmol/mg, and 1.23 nmol/mg correspondence to 10 nM, 50 nM, and 100 nM PMA concentration in comparison with non-treated HaCaT cells (0.49 nmol/mg). Furthermore, the results of Western blotting analysis showed that PMA treatment triggered the upregulation of MAP kinase pathway and activated the inflammation through NF-κB-mediated pathway (Figure 3K).

### 2.4. PMA Treatment Aggravated ROS Generation and Inflammatory Cell Infiltration on Zebrafish Larvae

**Figure 4:**
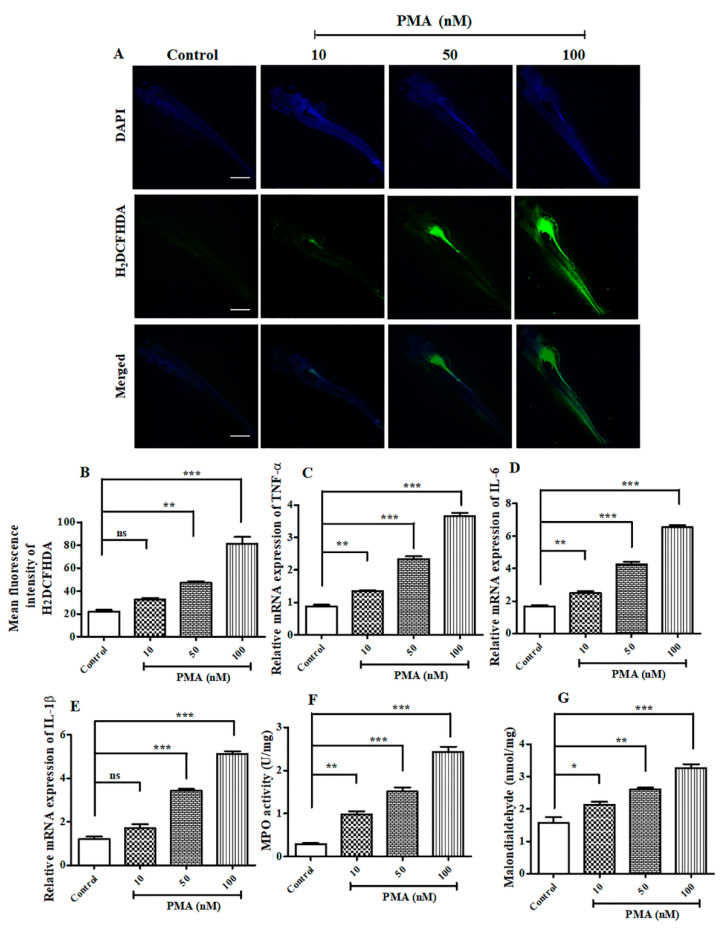
PMA induced elevated reactive oxygen species (ROS) and inflammatory response in Zebrafish larvae. (**A**) H_2_ DCFDA staining on Zebrafish larvae after PMA treatment on 6 dpf larvae, scale bar = 200 μM at 4× magnification (**B**) Mean fluorescence intensity of H_2_ DCFDA, (**C**) Relative mRNA expression of TNF-α, (**D**) Relative mRNA expression of IL-6, (**E**) Relative mRNA expression of IL-1β, (**F**) Myeloperoxidase assay, (**G**) Malondialdehyde assay. The data are represented as mean ± S.D. of three independent experiments * *p* < 0.05, ** *p* < 0.01, *** *p* < 0.001 and ns (non-significant). Control vs. PMA. Statistical significance analysis was carried out through a one-way analysis of variance (ANOVA) prism.

Since PMA is a potent cancer inducer and, consequently, increases inflammatory response, we decided to investigate the effect of PMA on ROS generation in 6 dpf zebrafish larvae. Consistent with the in vitro findings, the level of ROS generation exacerbated with increase in PMA concentration observed on 50 nM and 100 nM PMA treated groups (Figure 4A,B). Next, we evaluated the mRNA expression levels of important pro-inflammatory cytokines and observed a sharp dose-dependent increase on PMA treated groups (Figure 4C–E). Concurrently, we measured the activity of myeloperoxidase assay (MPO), which is abundantly expressed by neutrophils, granulocytes present on inflammatory response site [12]. Our results illustrated PMA treatment increased the expression of MPO (Figure 4F) dose-dependently (*p <* 0.0001), 10 nM (0.983 U/mg), 50 nM (1.51 U/mg), and 100 nM (2.43 U/mg). Furthermore, we also estimated the production of MDA, which is a major marker of oxidative stress. Consistent with in vitro findings, the levels of MDA (*p <* 0.0001) was remarkably upregulated (Figure 4G) with increased PMA dosage; indicating that PMA potently aggravated inflammatory response and ROS production in zebrafish larvae model.

### 2.5. Antioxidant Potential and Cytotoxic Effects of Tangeretin (TAN) on Immortalized Human Keratinocyte Cells

The structure of TAN is shown in Supplementary Figure S1A. We analyzed the antioxidant potential of TAN, which showed significant free radical scavenging potential through DPPH assay (Supplementary Figure S1B). The estimated IC_50_ value of TAN was found to be 39.93 μg/mL, and the IC_50_ of positive control i.e., ascorbic acid was 18.18 μg/mL in the assay. We also analyzed the cytotoxic potential of TAN on HaCaT cells. Consistent with the previous findings from others [13,14], and as observed through the MTT assay, the cell viability at 50 μM of TAN after 24 h exposure was found to be 81.06% (Supplementary Figure S1C), and did not exhibit higher toxicity assessed from LDH release (Supplementary Figure S1D), demonstrating that TAN exhibited lesser cytotoxic effect even at 50 μM against HaCaT cells. Moreover, it did not show any significant effect on the LDH production, HaCaT cell density, and morphology (Supplementary Figure S1E). The results revealed that TAN is not toxic to normal human keratinocyte at a particular dosage, and showed good antioxidant potential. So, we continued our experiment towards an in vivo ear inflammation model.

### 2.6. Tangeretin Counteracted PMA-Induced Epidermal Hyperplasia and Intra-Epidermal Neutrophilic Abscesses

**Figure 5:**
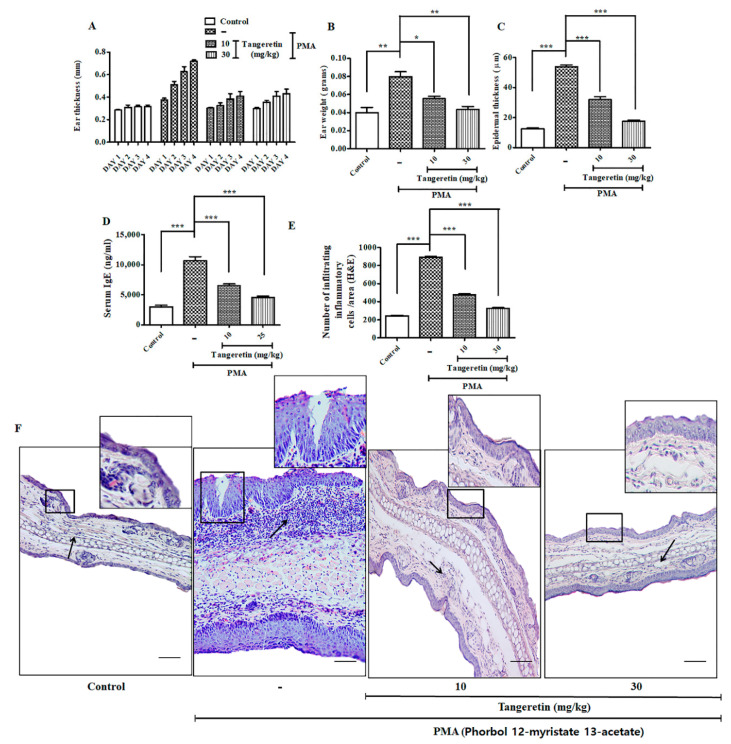
Tangeretin (TAN) remarkably ameliorated PMA induced epidermal hyperplasia intra-epidermal neutrophilic abscesses (mice, *n* = 5/group). (**A**) Ear thickness was measured every day after topical treatment of PMA and TAN for 3 days, (**B**) Ear weight of different mice group after sacrifice, (**C**) Epidermal thickness of mice ears measured through hematoxylin and eosin (H&E) staining data, (**D**) Serum IgE estimation from PMA treated mice, (**E**) Estimating the infiltrating inflammatory cells/area from H&E staining images, (**F**) Representative images of mice ear tissues after PMA induced inflammation measured through H&E staining. Square: the regions under the square represent massive re-epithelialization occurring after PMA treatment over a period of 3 days. Newly formed, thick epidermis as observed on PMA group. Black arrow: the marked regions on the images shows highly aggregated granulation tissue and an inflammatory cell rush comprising of scattered neutrophils, macrophages and other inflammatory cells. Scale bar (100 μM). The data are represented as mean ± S.D. of two independent experiments * *p* < 0.05, ** *p* < 0.01, *** *p* < 0.001 and ns (non-significant). Control vs. PMA, PMA vs. TAN 10 mg/kg and 30 mg/kg. Statistical significance analysis was carried out through a one-way analysis of variance (ANOVA) prism.

**Figure 6:**
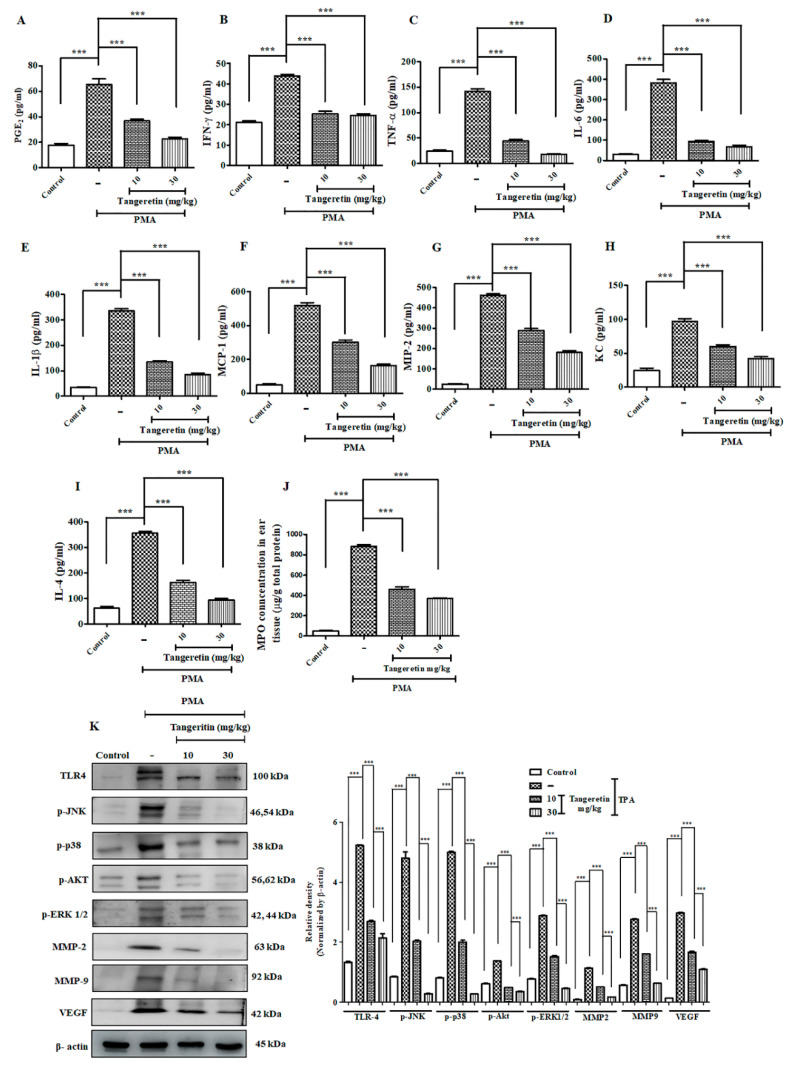
TAN exhibited potent anti-inflammatory response and blockaded cell proliferation pathway. Measurement of different inflammatory markers on mice after PMA treatment estimated by ELISA analysis. (**A**) PGE_2_, (**B**) IFN-γ, (**C**) TNF-α, (**D**) IL-6, (**E**) IL-1β, (**F**) Cox-2, (**G**) MCP-1, (**H**) MIP-2, (**I**) Keratinocyte chemoattractant (KC), (**J**) IL-4, (**K**) myeloperoxidase assay (MPO), Western blotting analysis on ear tissue homogenates showed that TAN was able to significantly reduce the inflammatory response challenged by PMA (10 μg/ear) and blockade the cell proliferation pathway validated. Densitometry analysis of these respective proteins were normalized by β-actin and evaluated through Image J software. The data are represented as mean ± S.D. of three independent experiments *** *p* < 0.001. PMA vs. control, TAN 10 mg/kg and 30 mg/kg vs. PMA. Statistical significance analysis was carried out through a one-way analysis of variance (ANOVA) prism.

For inducing skin inflammation, topical PMA administration (10 μg) was done on the mice ear for three consecutive days, which efficiently replicated the symptoms similar to human psoriasis in mice model [15]. PMA treated ears evidently had hyperkeratosis (epidermal thickening), parakeratosis (nucleated keratinocyte accumulates in the uppermost stratum corneum skin layer, and a greater neutrophilic intra-epidermal inflammatory cell infiltrate) (Figure 5F) [16]. TAN was able to significantly reduce the effect of edema and hyperplasia on the mice’s ear in a dose-dependent manner, as observed through ear images (Supplementary Figure S2A). Ear thickness and ear weight (*p* < 0.001) was also drastically reduced on the TAN groups compared to the PMA treated group (Figure 5A,B). Epidermal thickness score calculated from images obtained through hematoxylin and eosin (H&E) staining also revealed a positive counteracting effect of TAN, whereas only the PMA administration group revealed extended hyperkeratosis (Figure 5C). Additionally, the estimation of serum IgE (*p* < 0.0001) confirmed significant suppression (Figure 5D) in the TAN treated groups 10 mg/kg (6500 ng/mL) and 30 mg/kg (4533 ng/mL), compared to the PMA exposed group (10,667 ng/mL). Furthermore, we performed an infiltrating inflammatory cell counting (*p* < 0.0001) from H&E staining images and found a significantly inflammatory cell rush in the PMA treatment group, which subsided effectively on both doses of TAN treated groups (Figure 5E). Histological analysis (Figure 5F) revealed that TAN (10 mg/kg and 30 mg/kg) remarkably reduced the influx of inflammatory cells, granulation tissue accumulation, and decreased re-epithelialization, epidermal hyperplasia, in comparison to PMA groups. Similarly, we analyzed the important molecular markers of inflammatory cell infiltrations from the homogenized ear tissues demonstrated through ELISA. Our analysis confirmed the epidermal neutrophilic and macrophage recruitment through the estimation of the chemokines and cytokines mediating it. The result revealed TAN (10 mg/kg and 30 mg/kg) remarkably downregulated the production of major inflammatory markers, such as PGE_2_, IFN-γ, TNF-α, COX-2, IL-6, IL-1β, MCP-1, MIP-2, KC and IL-4 (Figure 6A–I); the concentration of the control group showed 16.91 pg/mL, 14.95 pg/mL, 24.106 pg/mL, 19.37 pg/mL, 25.27 pg/mL, 34.56 pg/mL, 46 pg/mL, 23.5 pg/mL, 23 pg/mL, and 63.33 pg/mL, respectively, whereas the PMA groups had an elevated expression of these inflammatory markers (62.87 pg/mL, 42.92 pg/mL, 144.13 pg/mL, 165.19 pg/mL, 375.01 pg/mL, 330.56 pg/mL, 527 pg/mL, 465.65 pg/mL, 99 pg/mL, and 355 pg/mL, respectively), which was significantly inhibited on both the treated groups of TAN 10 mg/kg (37.45 pg/mL, 24.11 pg/mL, 41.84 pg/mL, 61.94 pg/mL, 92.87 pg/mL, 137.17 pg/mL, 307.5 pg/mL, 282.5 pg/mL, 61.5 pg/mL, and 1633.33 pg/mL, respectively) and 30 mg/kg (23.26 pg/mL, 23.81 pg/mL, 17.61 pg/mL, 44.87 pg/mL, 64.50 pg/mL, 88.66 pg/mL, 167 pg/mL, 185 pg/mL, 41 pg/mL, and 94.33 pg/mL, respectively). MPO (*p* < 0.0001), is another important marker of neutrophil activation (Figure 6J) was noticeably highly upregulated in PMA group (882.5 5μg/g), which, upon TAN treatment (10 mg/kg; 460 μg/g and 30 mg/kg; 368.5 μg/g), was observably and significantly inhibited dose-dependently. Collectively, TAN treatment remarkably ameliorated PMA-induced epidermal hyperplasia and suppressed the expression of inflammatory cells.

### 2.7. Tangeretin Suppressed PMA-Induced Oxidative Stress and Boosted Antioxidant Enzyme Response

**Figure 7:**
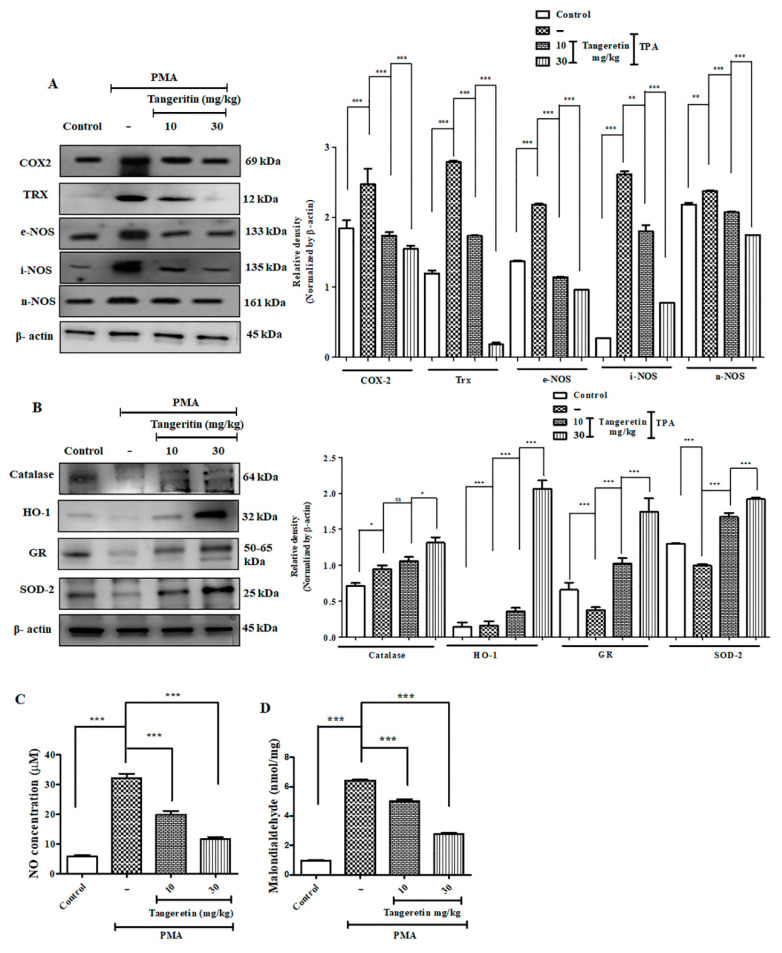
TAN neutralized PMA induced elevated ROS production by promoting antioxidant response. (**A**) Western blotting analysis of oxidative stress and inflammation markers evaluated from ear tissue homogenates, (**B**) Western blotting analysis of antioxidant activity markers evaluated from ear tissue homogenates, (**C**) Nitric oxide assay evaluated from mice ear tissue homogenates, (**D**) Malondialdehyde lipid peroxidation assay. Densitometry analysis of these respective proteins were normalized by β-actin and evaluated through Image J software. The data are represented as mean ± S.D. of three independent experiments * *p* < 0.05, ** *p* < 0.01, *** *p* < 0.001 and ns (non-significant). PMA vs. control, PMA vs. TAN 10 mg/kg and 30 mg/kg. Magnification = 200×. Statistical significance analysis was carried out through a one-way analysis of variance (ANOVA) prism.

TAN is a polymethoxylated flavonoid and has shown to possess high antioxidant activity [17], which directly participates in reduction of oxidative stress induced upon PMA-administration [18]. An excessive amount of endogenous ROS generation creates an imbalance in the redox reaction, consequently increasing the oxidative stress, which resultantly damages the important biomolecules necessary for cell survival [19]. TAN effectively downregulated the protein expression of COX-2 (*p* < 0.0293), TRX (*p* < 0.0001), e-NOS (*p* < 0.0001), i-NOS (*p* < 0.0001), and *n*-NOS (*p* < 0.0001) in a dose-dependent manner (Figure 7A). NO (*p* < 0.0042) and MDA (*p*< 0.0001) levels, respectively, are also shown in Figure 7C,D. The concentrations for control group were 5.41 μM and 0.97 nmol/mg, whereas PMA only treated groups expressed higher levels (30.785 μM and 6.425 nmol/mg), and TAN treated groups (10 mg/kg; 18.58 μM and 5.03 nmol/mg and 30 mg/kg; 10.92 μM and 2.775 nmol/mg) remarkably suppressed the expression of NO and MDA, which are major oxidative stress markers [20]. Subsequently, we also measured protein expression of antioxidant enzymes catalase (*p* < 0.0083), HO-1 (*p* < 0.0001), GR (*p* < 0.0001), SOD-2 (*p* < 0.0001) versus the PMA treated group that showed increased expression of TAN treatment groups (Figure 7B). Additionally, when an inflammatory stimuli activate the cells of the immune system, the different intracellular signaling pathway gets activated to carry the signal for activation of the inflammatory mediators such as cytokines TNF-α, IL-1β that acts through different surface receptors known as toll like receptors (TLRs), and activates the mitogen-activated protein kinases (MAPK) [21], thereby leading to the activation of NF-κB pathway. The inhibition of the MAP kinase pathway is one of the molecular targets for ameliorating inflammatory diseases [22].

We measured different inflammatory and cell proliferation pathway markers to observe TAN induced significant systematic downregulation of TLR-4 (*p* < 0.0001), *p*-JNK (*p* < 0.0001), *p*-p38 (*p* < 0.0001), *p*-ERK 1/2 (*p* < 0.0001), *p*-AKT (*p* < 0.0001), VEGF (*p* < 0.0001), MMP2 (*p* < 0.0001), MMP-9 (*p* < 0.0001) after TAN treatment, dose-dependently (Figure 6K). Altogether, TAN neutralized generation of free radicals to maintain a redox balance and normalized the elevated MAP kinase cell proliferation pathway induced by PMA exposure.

### 2.8. PMA Induced Nuclear Translocation and HIF-1α -NF-κB Crosstalk Kept at Bay by Tangeretin

**Figure 8:**
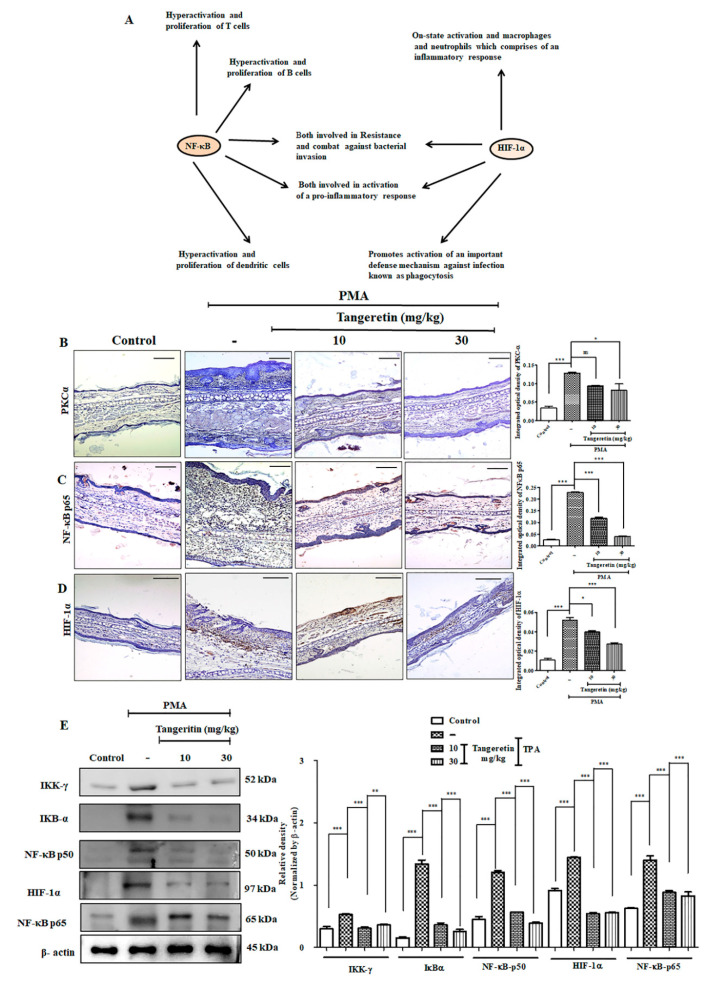
TAN blocks PMA induced hypoxia-inducible factor 1-alpha (HIF-1α) and nuclear factor kappa-light-chain-enhancer of activated b cells (NF-κB) inflammatory crosstalk. (**A**) Concept building diagram representing the interaction and crosstalk between HIF-1α and NF-κB after PMA challenge on mice ears, (**B**) PKCα expression on mice ear tissues evaluated by immunohistochemical analysis, (**C**) NF-κB-p65 levels evaluated by immunohistochemical analysis, (**D**) HIF-1α evaluated by immunohistochemical analysis, (**E**) Western blotting analysis cytoplasmic HIF-1α and NF-κB pathway markers evaluated from ear tissue homogenates. Scale bar (100 μM). Magnification = 200×. Statistical significance analysis was carried out through a one-way analysis of variance (ANOVA) prism. * *p* < 0.05, ** *p* < 0.01, *** *p* < 0.001 and ns (non-significant).

The interaction between HIF-1α and NF-κB (Figure 8A) has been observed in numerous diseases [23]. The crosstalk between them includes activating inflammatory cells, stimuli, regulators, and different targets, since both HIF-1α and NF-κB are transcription factors [24]. HIF-1α is also involved in regulating the action of NF-κB pathway activation in hypoxic neutrophil cell infiltration on inflamed tissue sites [25]. So, they are commonly involved in the activation of immune cells during inflammation. Western blotting analysis of hypoxia and NF-κB pathway markers revealed a statistically significant dose-dependent downregulation of HIF-1α (*p* < 0.0001), IKK-γ (*p* < 0.0001), IκBα (*p* < 0.0019), NF-κB-p50 (*p* < 0.0004), NF-κB-p65 (*p* < 0.0021) versus groups treated with PMA alone in a dose-dependent manner (Figure 8E). We further performed immunohistochemical staining on mice ear tissues for PKC-α (Figure 8B), NF-κB-p65 (Figure 8C), and HIF-1α (Figure 8D). PMA treatment groups had a more intense expression of the brown reaction product, which was observed to a lesser extent after TAN treatment.

**Figure 9:**
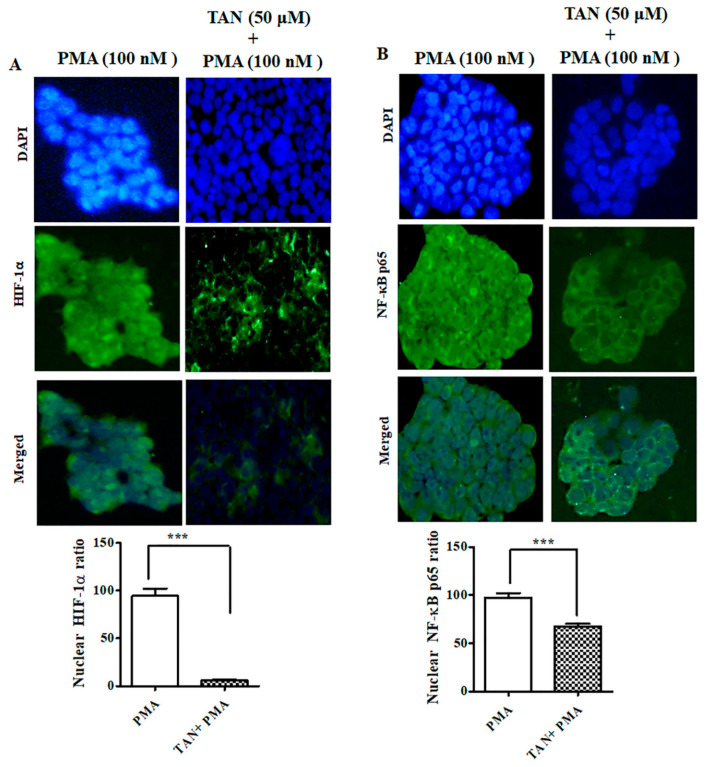
TAN inhibited PMA induced NF-κB-p65 and HIF-1α nuclear translocation on HaCaT cells. (**A**) Immunofluorescence staining of HIF-1α after treatment with tangeretin (50 μM, 24 h) and PMA (100 nM, 4 h), (**B**) Immunofluorescence staining of NF-κB-p65 after treatment with tangeretin (50 μM, 24 h) and PMA (100 nM, 4 h). Scale bar (100 μM). Magnification = 200×. *** *p* < 0.001.

In addition, we performed immunofluorescence staining on HaCaT cells after treatment with TAN (50 μM, 24 h) and PMA (100 nM, 4 h). Our results revealed, PMA effectively translocated HIF-1α and NF-κB-p65 observed through accumulated green fluorescence intensity in the nuclear region. However, TAN remarkably inhibited the nuclear translocation of HIF-1α and NF-κB-p65 in HaCaT cells, as shown in Figure 9A,B. Taken together, these results substantiated the role of TAN to blockade the interaction of two important transcription factors involved in hypoxia and inflammation in vitro and in vivo.

## 3. Materials and Methods

### 3.1. Chemicals

Tangeretin (TAN) was purchased from Cayman Chemicals (Cay10009911-100). Fetal bovine serum (Gibco), DMEM, PMA (phorbol 12-myristate 13-acetate), penicillin-streptomycin, Trypsin, DAPI (4′,6-diamidino-2-phenylindole), 3-(4,5-dimethylthiazol-2-yl)-2,5-diphenyltetrazolium bromide (MTT), dimethyl sulfoxide (DMSO), dichlorodihydro-fluorescein diacetate (H_2_ DCFDA), Rhodamine 123, Hoechst was purchased from Sigma-Aldrich, St. Louis, MO, USA. Other chemicals and reagents used for the experiment but not mentioned here were purchased from the highest grade available used for molecular studies. The complete list of the primary and secondary antibodies used in this study is provided in Supplementary Tables S1 and S2.

### 3.2. Allium cepa Genotoxicity Analysis

*Allium cepa* bulbs were cultivated in the laboratory by placing the bulbs in a 100 mL beaker, filled with tap water and allowed to grow for 3–4 days. Next, the bulbs were treated with (1 μg, 3 μg, 10 μg) PMA for 24 h. The rootlets were collected, fixed in Carnoy’s fixative, and immersed in 5 M HCl (20 min) for proper hydrolysis, washed with tap water and stained with acetocarmine stain (10 min), washed with taper water and covered with a cover slip and observed under the microscope for chromosomal aberrations [26]. The slides were examined for any mitotic changes at 1000×. Cell division was analyzed by counting 300 cells per bulb after 24 h treatment with PMA and mitotic index was calculated.

(1) $$\mathrm{Active}\;\mathrm{mitotic}\;\mathrm{index}\;(\mathrm{AMI})\;\%\;=\frac{\mathrm{Total}\;\mathrm{number}\;\mathrm{of}\;\mathrm{dividing}\;\mathrm{cells}}{\mathrm{Total}\;\mathrm{number}\;\mathrm{of}\;\mathrm{counted}\;\mathrm{cells}}\times \;100$$

(2) $$\mathrm{Total}\;\mathrm{abnormality}\;\mathrm{Percentage}\;\%\;=\frac{\mathrm{Total}\;\mathrm{number}\;\mathrm{of}\;\mathrm{abnormal}\;\mathrm{cells}}{\mathrm{Total}\;\mathrm{number}\;\mathrm{of}\;\mathrm{cells}\;\mathrm{counted}}\times \;100$$

### 3.3. Zebrafish Husbandry and PMA Toxicity Analysis

Wild type zebrafish (AB strain) were allowed to grow and reproduce under healthy environmental conditions (28 °C and 80% humidity). Zebrafish were adjusted to 14 h light and 10 h dark cycle. The next day, fertilized eggs were collected within 4 h post fertilization (hpf). Healthy zebrafish embryos (*n* = 20/group) were properly maintained at a suitable temperature (28 °C) in E3 media containing (0.17 mM KCl, 5 mM NaCl, 0.16 mM MgSO_4_,0.4 mM CaCl_2_, 0.1% methylene blue) as described [27,28]. PMA treatment (0–100 nM) was started from 1 day post fertilization (dpf) to 120 dpf. All the dead embryos and larvae during the experiment was removed and discarded every 12 h. Images were collected throughout the experimental period to check for abnormalities.

### 3.4. DPPH Assay

The potential of TAN to scavenge free radical was analyzed by using 1,1-Diphenyl-2-picrylhydrazyl (DPPH) at 0.1 μM, and dissolved in absolute ethanol [29]. The biochemical reaction was performed in a 96-well assay plate, where ascorbic acid was taken as a positive control and the different concentrations of TAN was mixed to 200 μL freshly prepared DPPH solution. The mixture was vigorously shaken and allowed to incubate in a dark room for 30 min. Finally, the absorbance of the different samples and positive control used for the experiment was measured using a spectrophotometer (UV-2120 Optizen, Daejeon, Korea) 517 nm. The potential of the used compound to scavenge the free radical was calculated using the following formula mentioned below:

(3) $$\mathrm{Scavenging}\;\mathrm{ability}\;\%\;=1-\frac{A_{t}}{A_{0}}\times 100X$$

where A*_t_* sis the absorbance reading of the samples and A_0_ represents the absorbance reading of the blank.

### 3.5. Cell Culture Studies and Toxicity Analysis

Immortalized human keratinocyte (HaCaT, passage number 2, ATCC) cells were cultured in a DMEM media, supplemented with 10% FBS and 1% penicillin-streptomycin on a CO_2_ incubator with temperature maintained at 37 °C. For performing in vitro experiments, HaCaT cells were treated with PMA or TAN (alone) at a varying concentration to evaluate the cell viability and toxicity. To study the cytotoxic effect on HaCaT cells, we carried out the cell viability MTT assay. Firstly, the cells were seeded in a 96 well plates at a density of 1 × 10^6^ cells/mL and allowed to adhere and grow in a CO_2_ incubator. Depending on the amount of time it takes for the cells to reach around 70% confluency, approximately 48 h, the cells were treated with different concentrations of TAN, PMA, or TAN+ PMA. After 24 h of incubation, the old media was discarded, and the cells were washed with 100 μL of PBS. After washing, 100 μL of DMEM media was added to each well, along with 10 μL of MTT (5 mg/mL) and incubated for 4 h at RT. Later, the MTT solution was aspirated out carefully and to each well 50 μL of DMSO (dimethyl sulfoxide), and was kept in an ELISA plate shaker covered with aluminum foil and incubated for 30 min at RT for dissolving the formed formazan crystals. Later, the OD value was measured at 550–570 nm, using an ELISA plate reader (TECAN 200 infinite PRO, Männedorf, Switzerland). Whereas, to perform LDH assay, we used a commercially available kit (Abbkine, CAT# KTA1030, Wuhan, China).

### 3.6. Hoechst-33342 Staining for Detection of Nuclear DNA Condensation

For performing Hoechst 33342 staining, we followed the previously published protocol [30]. After incubation with different doses of PMA (10 nM, 50 nM, 100 nM), the cells were washed with PBS (2 times) and allowed to be fixed by treatment with ice cold 4% formaldehyde. The cells were washed in PBS and incubated with Hoechst 33342 (1 μg/mL) for 15 min at 37 °C in a CO_2_ incubator. After PBS washes, the cells were observed using an Olympus BX50 (Feasterville, PA, USA) fluorescence microscope at 200× magnification.

### 3.7. PMA-Induced Ear Inflammation

BALB/c male mice around 4–6-weeks old were purchased from the Orient Bio, Inc. (Seoul, Korea). All mice were caged and stored under a 12 h light and dark cycle in a temperature-humidity controlled in the animal breeding center of Advanced Bio convergence center of Pohang Technopark foundation, South Korea at a temperature of 19–22 ± 2 °C, and a relative humidity of 50 ± 5%, allowed to acclimate for some few days with free access to drinking water and rodent food. All animal-related experiments were conducted after obtaining the approval of the Pohang Technopark Laboratory Ethics Committee on 06.30.2018 bearing the number ‘ABCC2018004′.

Ear inflammation was induced on both the ears (*n* = 5 mice/group) by topically treating PMA as per the previously published protocol [31]. PMA (10 μg) was applied the mice’s ears every 24 h for 3 days. After 1 h of PMA treatment, TAN (10 mg/kg and 30 mg/kg) was topically treated onto the ears of each mouse. Next, after 1 h of TAN treatment, we measured the ear thickness using a vernier caliper. On the final day of the experiment, all experimental mice were anaesthetized using isoflurane liquid inhalation and sacrificed and the whole ear weight was measured. For histological analysis, an 8 mm diameter punch from the mice’s inflamed region of the ear was collected and stored in 4% paraformaldehyde (PFA) and fixed, to prevent decaying of tissues until further experimentation.

### 3.8. Hematoxylin and Eosin Staining

For H&E staining, we followed the protocol from previously published paper [32]. Paraffin embedded tissues were sectioned at a thickness of 6–8 μM, using a histocore rotary microtome. The slides were analyzed under a light microscope (200× resolution).

### 3.9. Western Blotting

Mice ear tissue lysate were prepared by homogenizing the ear tissue and adding 400 μL of RIPA buffer (1:100 dilutions of protease and phosphatase inhibitor) for 20 mg of ear tissue. The tissues were vortexed vigorously and incubated for 15 min at −20 °C. Next, the tissues were centrifuged at 12,000× *g* (4 °C) for 10 min. The concentration of the protein was measured through a Bradford assay at 595 nm. The proteins were loaded onto an SDS-Polyacrylamide gel, according to the previously published paper [33], and analyzed the protein of interest.

### 3.10. Quantitative Determination of Inflammatory Markers through ELISA Assay

For performing ELISA assay as previously mentioned [34,35]. Briefly, we homogenized the cells/tissues with a RIPA buffer, which were incubated on ice for 15 min. The homogenates were further centrifuged at 12,000× *g* for 10 min. After centrifugation, the supernatant was collected and IL-4, IL-6, IFN-γ, PGE2, TNF-α, MIP-2, IL-1β, MCP-1, KC, and Cox-2 were measured from the supernatants by ELISA technique, according to manufacturer’s protocol (R&D Systems Quantikine ELISA kits, Minneapolis, MN, USA). The detailed list of the ELISA kits used in the experiment is provided in Supplementary Table S3.

### 3.11. Myeloperoxidase Activity Assay

For the estimation of myeloperoxidase (MPO) activity on homogenized cell/tissues, we used a TMB substrate solution. Firstly, 10 μL of ear sample homogenates was added to 80 μL of H_2_O_2_ (0.75 mM) and 110 μL of TMB solution [36]. After gently mixing the solution, the 96 well microliter plates were incubated for 5 min at 37 °C. We added 50 μL of 2 M H_2_SO_4_ to stop the reaction in the solution. Finally, OD value absorbance was measured by using a microtiter plate reader (Bio-Tek Instrument Co., Winooski, VT, USA) at 450 nm.

### 3.12. Nitric Oxide Assay

For measuring the concentration of nitrite, we used Griess Reagent (Sigma, Spruce street, St. Louis, MO, USA). The different concentrations of homogenized samples were prepared in 1 mL of PBS. After that, 0.5 mL of 10 mM sodium nitroprusside reagent was added onto each sample and allowed to react for 3 h at RT. After incubation, an equal volume of Griess reagent was added and incubated for 10 min. The intensity of the pink color formation was measured through spectrophotometer (UV-2120 Optizen, Mecasys, Daejeon, Korea) at 540 nm. For the determination of the quantity of nitrite, a standard curve was plotted with different concentration of sodium nitrite (Thermofisher, Waltham, MA, USA). We used the standard curve for determining the concentration of nitrite in the different samples.

### 3.13. Malondialdehyde Assay for Estimation of Lipid Peroxidation

For estimating lipid peroxidation, we measured the concentration of malondialdehyde (MDA) from the homogenized samples, following the manufacturer’s protocol (Abcam). Optical density was measured at 532 nm using an ELISA plate reader (TECAN 200 infinite PRO, Männedorf, Switzerland).

### 3.14. Immunohistochemistry

For performing immunohistochemistry analysis. Firstly, the slides were heated for antigen retrieval step and dewaxing step with xylene twice (5 min). Slides were transferred to 100%, 95%, 80%, 70%, 50% EtOH for dehydration (5 min), washed with PBS (3 times) and incubated with blocking buffer solution (SuperBlock, Thermofisher Scientific). Later, slides were treated with primary antibodies and incubated for 3 h at RT. Next, the tissues were blocked again with 3% H_2_ O_2_ for 15 min to suppress the endogenous peroxidase activity. Slides were PBS washed and incubated with secondary antibody for 1 h at RT. Subsequently, DAB substrate solution was added to each slide and incubated (15 min). Next, the slides were washed with distilled water and treated with hematoxylin (3 min) for counterstaining. Finally, slides were dehydrated, dewaxed, and fixed with a mounting medium, allowed to air dry, and observed under a microscope (200×) (Olympus BX50 Fluorescence Microscope, Bustleton Pike Feasterville-Trevose, PA, USA).

### 3.15. Intracellular ROS Generation Assay

For the detection of intracellular reactive oxygen species (ROS) production, we referred to the previously published paper [37]. HaCaT cells and zebrafish larvae were by incubated with H_2_ DCFDA (10 μM) for 30 min at 37 °C. The ROS production was analyzed using an Olympus BX50 fluorescence microscope at 200× magnification.

### 3.16. Immunofluorescence Staining

For performing immunofluorescence staining, HaCaT cells were seeded in glass cover slips at a density of 2 × 10^4^/well and allowed to adhere and grow. After treatment with PMA and TAN, the cells were fixed using 4% paraformaldehyde (10 min) at RT. Next, the cells were washed three times with ice cold PBS (3 min). Antigen retrieval step was performed by heating coverslips at 95 °C (10 min), using antigen retrieval buffer. Next, slides were washed with PBS followed by incubation with 0.1% triton X-100 (10 min) for permeabilization. Next, the cells were treated with blocking buffer (1% BSA in PBST) for 30 min. Later, the cells were incubated with primary antibodies (HIF-1α and NF-κB p65) overnight at 4 °C. Further, we washed the cells with PBS and incubated with FITC-secondary antibody for 1 h at RT, followed by counterstaining with DAPI (0.1–1 μg/mL) for 1 min. Finally, the coverslips were placed onto slides and sealed with mounting medium and observed under Olympus BX50 fluorescence microscope (200×).

### 3.17. RT-PCR Analysis

For performing RT-PCR, 10 zebrafish embryos were pooled together in Tri-Reagent (Sigma, USA), according to their respective groups. Further cDNA was prepared using iScript cDNA synthesis kit (Bio-Rad, Lincoln Centre Drive, Foster City, CA, USA), in order to perform reverse transcription. Quantitative PCR was performed with the Go Taq qPCR Mastermix (Promega, Woods hollow road, Madison, WI, USA) using a CFX96 Real-Time PCR thermocycler (Bio-Rad, Lincoln Centre Drive, Foster City, CA, USA). We used Tubulin-1 as a housekeeping gene for comparing the fold change in expression of mRNA. Finally, the data were examined using ΔΔCt method. The details of all the primers used is described in detail in Supplementary Table S4.

### 3.18. Statistical Significance Analysis

For determining the quantitative results of the experiments performed in this study, the values are expressed as a mean ± standard error (SD). The statistical significance and the differences in the experimental groups were calculated by using one-way analysis of variance (ANOVA) with Tukey’s test comparing all pair of columns, and Dunnett’s post-comparison test for multiple groups; where * represents *p*-values < 0.05, ** represents *p*-values < 0.01, and *** represents *p*-values < 0.001.

## 4. Conclusions

Numerous toxicity studies on different kinds of phorbol esters have been widely carried out on different model system. PMA is one such phorbol ester that is extensively studied for its role as a potent inflammation inducer and tumor promoter. A toxicity study of PMA on different model system such as *Allium cepa* test was widely used for studying the cytotoxic and genotoxic potential of substances. This test is also used for evaluating the mutagens and detecting toxic substances found in the environment which directly contributes to preventing environmental toxicity [38]. In the current study, we recorded a significant inhibition in the root growth, chromosomal aberrations, and mitotic dysfunction on the onion root tips, indicating a manifestation of arrest in cell division after 24 h of PMA treatment. The zebrafish embryotoxicity test is considered a suitable assessment of toxicity study for different kinds of pollutant [39]. We observed that PMA exerted severe and adverse morphological deformities that were aggravated with increased PMA dose and prolonged exposure. The hatching rate and survivability rate severely dropped, indicating the harmful effects of PMA on zebrafish growth and development. Our study on the immortalized human keratinocyte (HaCaT) cell line exhibited severe morphological distortions and cell viability dropped drastically with prolonged PMA exposure (Figure 3B). PMA treated on mice ear triggered a massive influx of inflammatory cells on the region of PMA treated tissues. Through histological staining, we were able to identify excessive aggregation of the granulation tissues, polymorphonuclear leukocytes (PMN) accumulation, and an inflammatory cell rush comprising of scattered inflammatory cells. In summary, toxicity studies revealed PMA to be an environmental toxin, causing highly inflammatory state resembling a human psoriasis model, morphological deformities, chromosomal mutations, and cell death in vitro and in vivo.

**Figure 10:**
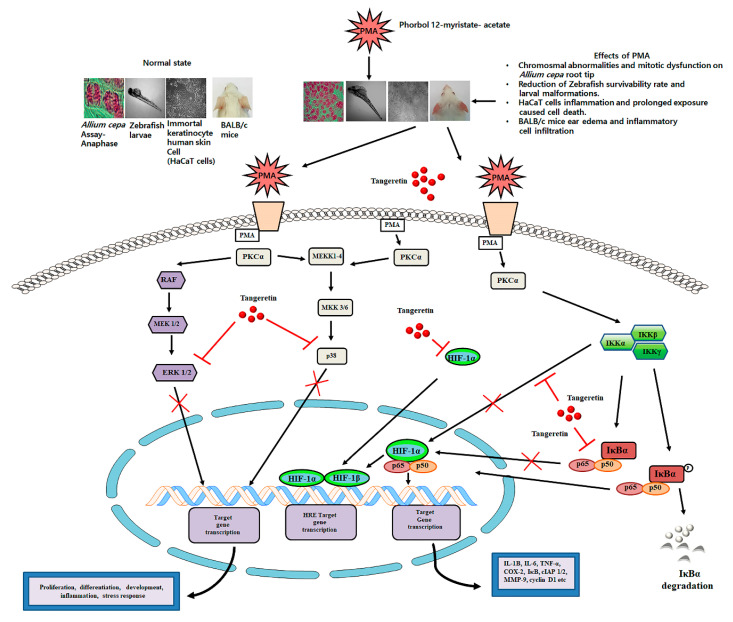
A schematic illustration of the role of tangeretin in counteracting PMA-induced inflammatory response on in vitro and in vivo model systems.

PMA has been well-known to enhance ROS production through the activation of inflammatory pathways and also decline in the enzymes involved in ROS detoxification process. PMA has also been shown to inhibit and reduce the basal level of SOD, catalase, and glutathione peroxidase, which, according to our study, showed significant upregulation of these antioxidant enzymes after treatment with TAN. As we know, ROS and inflammation are intertwined in various diseases. Numerous diseases have an associated hyperactivated inflammatory signaling response, dysfunctional cellular metabolism, and a modified redox balance suited for inflammatory pathways [40]. The toxicity of TAN on the murine model was relatively miniscule, and did not exert toxic effects, even at a dose of 3 g/kg [13]. TAN has been found to be inhibiting the activity of PKC activated by PMA [41]. We observed TAN treatment blockaded the degradation of IκBα and subsequently inhibiting the translocation of NF-κB- p65 and HIF-1 α to the nucleus, also observed in previous studies on different cell lines [42]. A schematic representation is provided in Figure 10. The inflammatory response, ear edema, and hyperplasia were subsequently subdued on TAN treatment group mice. Consistent with the findings from other studies, we also conclude that TAN can be used as an anti-inflammatory agent on an in vitro and in vivo model system, without exhibiting lethal toxicity at lower doses.

## Supplementary Materials

Supplementary materials can be found at https://www.mdpi.com/1422-0067/21/23/9261/s1.
