# Supplementary material for: Phorbol 12-Myristate 13-Acetate Induced Toxicity Study and the Role of Tangeretin in Abrogating HIF-1α-NF-κB Crosstalk In Vitro and In Vivo
Source: Int J Mol Sci. 2020 Dec 4;21(23):9261. doi: 10.3390/ijms21239261 (PMC7729754; doi:10.3390/ijms21239261)
Supplement: Supplementary file 1 [file ijms-21-09261-s001.pdf]

Supplementary information

Phorbol 12-Myristate 13-Acetate Induced Toxicity Study and the Role of Tangeretin in Abrogating HIF-1α-NF-κB Crosstalk *In vitro* and *In vivo*

Sukkum Ngullie Chang [1,2,†], Debasish Kumar Dey [1,†], Seong Taek Oh [2,3,†], Won Ho Kong [2], Kiu Hyung Cho [4], Ebtesam M. Al-Olayan [5], Buyng Su Hwang [6], Sun Chul Kang [1,*] and Jae Gyu Park [2,*]

[1] Department of Biotechnology, Daegu University, Gyeongsan, 38453, Republic of Korea

[2] Advanced Bio Convergence Center, Pohang Technopark Foundation, Pohang, Gyeongbuk 37668, Republic of Korea

[3] Okinawa Research Center Co. Ltd,13-33, Suzaki, Uruma-si, Okinawa Ken, 904-2234, Japan

[4] Research group, Gyeongbuk Institute for Bio Industry (GIB), Andong, 36728, Republic of Korea

[5] Department of Zoology, Faculty of Science, King Saud University, Riyadh 11451, Saudi Arabia

[6] Nakdonggang National Institute of Biological Resources, Sangju, 37242, Republic of Korea

[†] Contributed equally to this work

*Corresponding authors: Email: sckang@daegu.ac.kr (S.C.K.); jaegpark@gmail.com (J.G.P.); Fax: +82-53-850-6569 (S.C.K.); Fax: +82-54-223-2780 (J.G.P.)

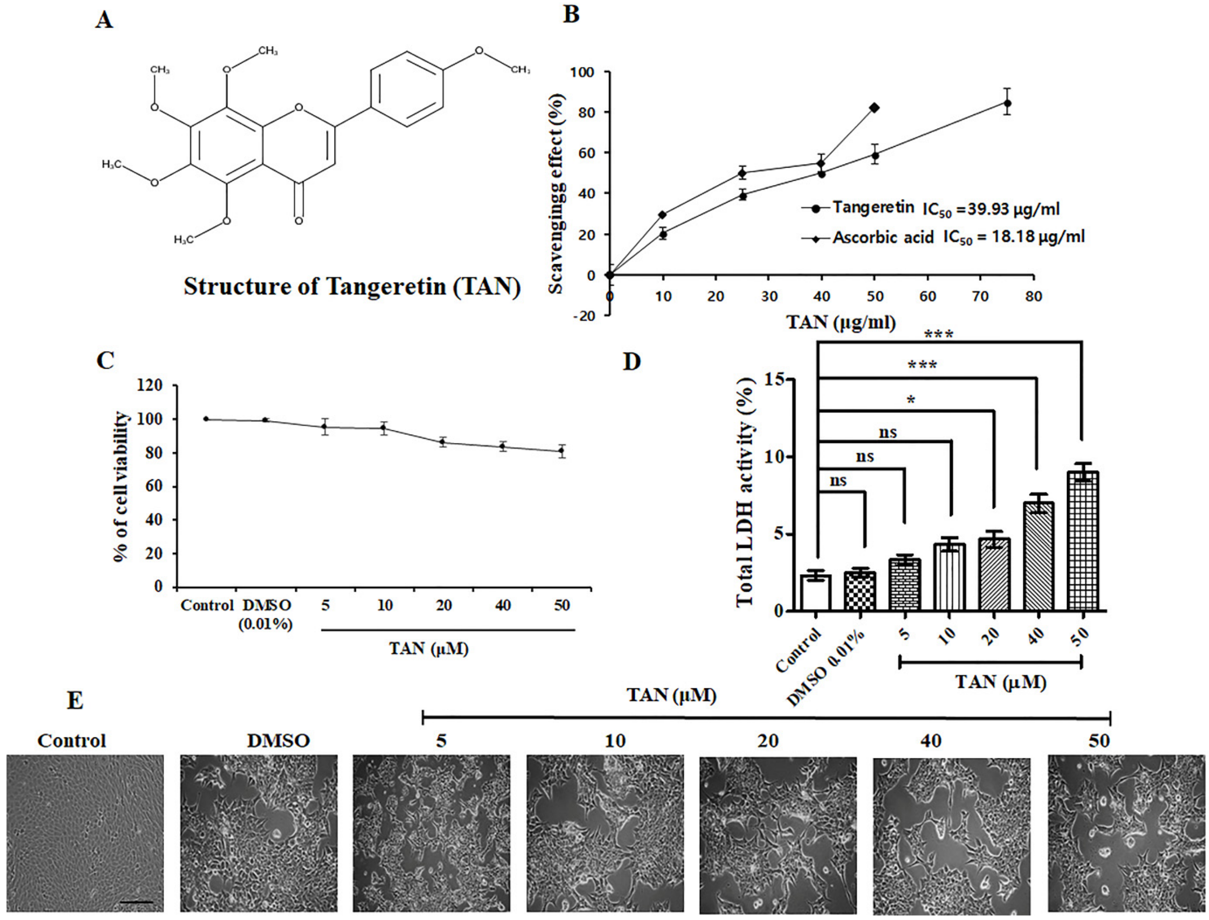

Figures S1. TAN exhibited high free radical scavenging potential and low toxicity on HaCaT cells (**A**) Structure of TAN (**B**) DPPH assay to evaluate the free radical scavenging property of TAN (**C**) Cytotoxic evaluation of TAN estimated through MTT assay on immortalized human keratinocyte (HaCaT) cells (D) LDH cytotoxicity assay (**E**) Morphological image of HaCaT cells captured after 24h of treatment with TAN at different concentration (5, 10, 20, 40, and 50 µM) and highest dose of TAN had a DMSO (0.01%) control (no treatment). Scale bar (100 µm). The data are represented as the means ± S.D. of three independent experiments *$p< 0.05$, **$p<0.01$, ***$p< 0.001$. Statistical significance analysis was carried out through one-way analysis of variance (ANOVA) prism.

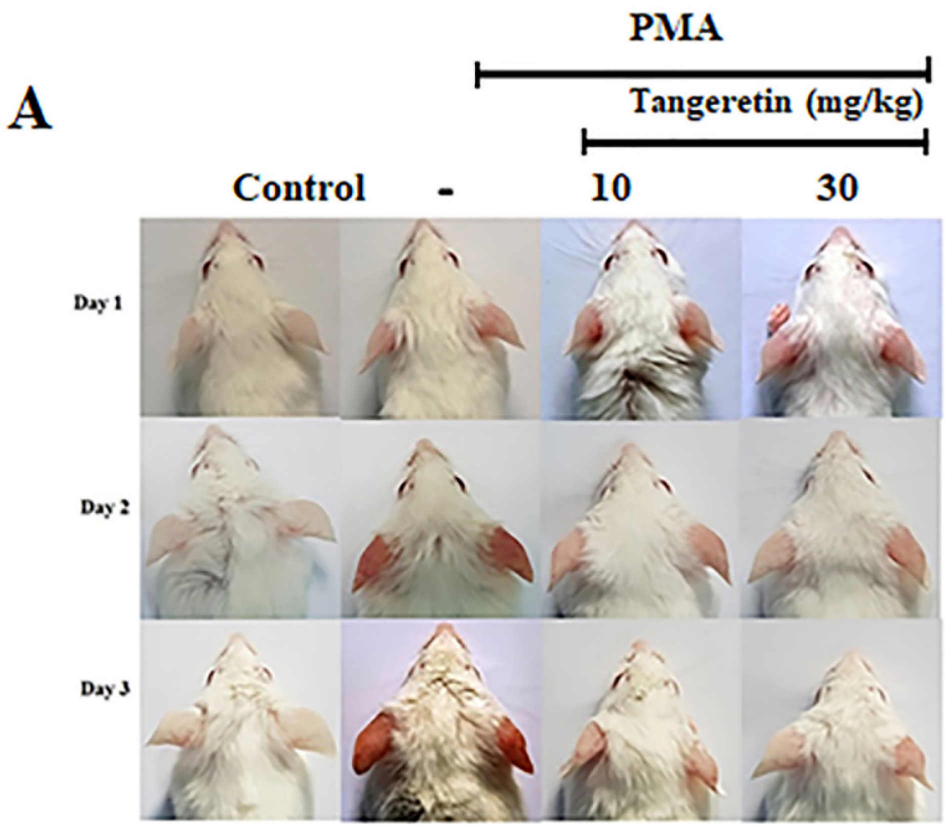

Figure S2. Mice ear images captured over a period of 3 days after topical administration of PMA and TAN treatment.

Table S1. List of primary antibodies used in the study.

S. No.	Protein name	Company name	Molecular weight	Dilution	Host	Secondary antibody
1	iNOS (ab3523)	Abcam	135	1:200	Rabbit	Goat anti rabbit-HRP
2	eNOS (ab66127)	Abcam	133	1:1000	Rabbit	Goat anti rabbit-HRP
3	nNOS (#4234)	Cell signaling	161	1:1000	Rabbit	Goat anti rabbit-HRP
4	COX-2 (#4842)	Cell signaling	69	1:1000	Rabbit	Donkey anti goat-HRP
5	TNF-α (#3707)	Cell signaling	26	1:1000	Rabbit	Goat anti rabbit-HRP
6	p-ERK 1/2 (sc-16982)	Santa cruz	42/44	1:50-1:500	Rabbit	Donkey anti goat-HRP
7	p-p38 (sc-17582-R)	Santa cruz	38	1:50-1:500	Rabbit	Goat anti rabbit-HRP
8	p-JNK (SC-12882-R)	Santa cruz	46,54	1:100-1:1000	Rabbit	Goat anti rabbit-HRP
9	TLR4 (BS3489)	Bioworld	100	1:100-1:1000	Rabbit	Goat anti rabbit-HRP
10	p-Akt (SC-7985-R)	Santa cruz	62,56	1:1000	Rabbit	Donkey anti goat-HRP
11	Trx (SC-20146)	Santa cruz	12	1:1000 1:5000	Rabbit	Goat anti rabbit-HRP
12	GR (SC-32886)	Santa cruz	50-65	1:100-1:1000	Goat PC	Donkey anti goat-HRP
13	HO-1 (SC-10789)	Santa cruz	32	1:100-1:1000	Rabbit	Goat anti rabbit-HRP
14	MMP-2 (SC-10736)	Santa cruz	63,72	1:100-1:1000	Rabbit	Goat anti rabbit-HRP
15	MMP-9 (SC-6840)	Santa cruz	92	1:100-1:1000	Rabbit	Goat anti rabbit-HRP
16	VEGF (SC-152)	Santa cruz	21,42	1:100-1:1000	Rabbit	Goat anti rabbit-HRP
17	IKKγ (#8517)	Cell signaling	52	1:1000	Rabbit	Donkey anti goat-HRP
18	IκBα (BS90963)	Bioworld	34	1:1000 1:5000	Rabbit	Goat anti rabbit-HRP
19	NF-κB p50 (SC-114)	Santa cruz	50	1:100-1:1000	Rabbit	Goat anti rabbit-HRP
20	NF-κB p65 (#8242)	Cell signaling	65	1:100-1:1000	Rabbit	Goat anti rabbit-HRP
21	Catalase (sc-50508)	Santa cruz	64	1:100-1:1000	Rabbit	Goat anti rabbit-HRP
22	SOD-1 (sc-11407)	Santa cruz	23	1:100-1:1000	Rabbit	Goat anti rabbit-HRP
23	SOD-2 (sx-30080)	Santa cruz	25	1:100-1:1000	Rabbit	Goat anti rabbit-HRP
24	HIF-1α (#NBP1-02160)	Bioworld	97	1:1000	Rabbit	Goat anti rabbit-HRP
25	PKC-α (Sc-8393) (#4967)	Santa cruz s	85	1:50-1:500	Mouse	Goat anti mouse-HRP
26	β-actin (#4967)	Cell signaling	45	1:1000	Rabbit	Goat anti rabbit-HRP

Table S2. List of secondary antibodies used in the study.

S. No.	Name of secondary antibody	Company name	Dilution	Reactivity
1	Goat anti rabbit-HRP (NBP2-30348H)	Novus Biologicals (Bethyl)	1:5000	Anti-rabbit
2	Donkey anti goat-HRP (NBP2-68552)	Novus Biologicals (Bethyl)	1:5000	Anti-goat
3	Goat anti mouse-HRP	Santa cruz	1:2000-1:10000	Anti-mouse

Table S3. List of ELISA assay kits used in the study.

S. No.	Name of secondary antibody	Company name
1	Mouse IL-1β/IL-1F2 (#MLB00C)	Quantikine ELISA
2	Mouse IL-6 (#M6000B)	Quantikine ELISA
3	Mouse TNF-α (#MTA00B)	Quantikine ELISA
4	Mouse IFN-γ (#MIF00)	Quantikine ELISA
5	Mouse PGE_2 (#KGE004B)	Quantikine ELISA
6	Mouse/Rat CCL2/JE/MCP-1 (MJE00)	Quantikine ELISA
7	Mouse CXCL2/MIP-2 (DY452-05)	Quantikine ELISA
8	Lipid Peroxidation (MDA) Assay kit (ab118970)	Abcam
9	Mouse CXCL1/KC Quantikine ELISA Kit (MKC00B)	Quantikine ELISA
10	Human IL-6 Quantikine ELISA Kit (D6050)	Quantikine ELISA
11	Human IL-1 beta/IL-1F2 Quantikine ELISA Kit (DLB50)	Quantikine ELISA
12	Human TNF-alpha Quantikine ELISA Kit (DTA00D)	Quantikine ELISA
13	LDH cytotoxicity Assay Kit (CAT# KTA1030)	Abbkine

Table S4. List Primers used in the study.

Primers	5'-3' sequence	Accession number in GenBank
TNF-α F1	CTGCTTCACGCTCCATAAGA	AY427649.1
TNF-α R1	GCCTGGTCCTGGTCATCTC	AY427649.1
IL−6F1	GGTGAGAGACGGAGAGATGGAT	JN698962.1
IL−6R1	CACGCTGGAGAAGTTGAACAG	JN698962.1
IL−1βF1	ACAGAATGAAGCACATCAAACC	AY340959.1
IL−1βR1	ACAGAATGAAGCACATCAAACC	AY340959.1
